# Optimizing the protocol for modified natural cycle frozen embryo transfer (mNC-FET): a multicentre, single-blinded randomized controlled trial

**DOI:** 10.1093/hropen/hoag003

**Published:** 2026-01-13

**Authors:** Marte Saupstad, Clara Colombo, Sara Johanna Bergenheim, Nina Pistoljevic-Kristiansen, Tine Vrist Dam, Jeanette Wulff Bogstad, Lisbeth Prætorius, Nina la Cour Freiesleben, Anna Klajnbard, Birgitte Oxlund-Mariegaard, Peter Humaidan, Ellen Christine Leth Løkkegaard, Merete Husth, Ulla Breth Knudsen, Anette Gabrielsen, Julie Lyng Forman, Morten Rønn Petersen, Kristine Løssl, Anja Pinborg

**Affiliations:** Fertility Clinic, Department of Gynaecology, Fertility and Obstetrics, Copenhagen University Hospital—Rigshospitalet, Copenhagen, Denmark; Fertility Clinic, Department of Gynaecology, Fertility and Obstetrics, Copenhagen University Hospital—Rigshospitalet, Copenhagen, Denmark; Fertility Clinic, Department of Gynaecology, Fertility and Obstetrics, Copenhagen University Hospital—Rigshospitalet, Copenhagen, Denmark; Fertility Clinic, Department of Gynaecology, Fertility and Obstetrics, Copenhagen University Hospital—Rigshospitalet, Copenhagen, Denmark; Fertility Clinic, Department of Gynaecology, Fertility and Obstetrics, Copenhagen University Hospital—Rigshospitalet, Copenhagen, Denmark; Fertility Clinic, Department of Gynaecology, Fertility and Obstetrics, Copenhagen University Hospital—Rigshospitalet, Copenhagen, Denmark; Fertility Clinic, Department of Obstetrics and Gynaecology, Copenhagen University Hospital Hvidovre, Hvidovre, Denmark; Fertility Clinic, Department of Obstetrics and Gynaecology, Copenhagen University Hospital Hvidovre, Hvidovre, Denmark; Department of Clinical Medicine, University of Copenhagen, Copenhagen, Denmark; Fertility Clinic, Department of Obstetrics and Gynaecology, Copenhagen University Hospital Herlev, Herlev, Denmark; Fertility Clinic, Department of Obstetrics and Gynaecology, Zealand University Hospital Koege, Koege, Denmark; Skive Regional Hospital, University Clinic for Fertility, Skive, Denmark; Department of Clinical Medicine, Aarhus University, Aarhus, Denmark; Department of Obstetrics and Gynaecology, Copenhagen University Hospital North Zealand, Hilleroed, Denmark; Fertility Clinic, Department of Gynaecology and Obstetrics, Aalborg University Hospital, Aalborg, Denmark; Department of Clinical Medicine, Aarhus University, Aarhus, Denmark; The Regional Hospital Horsens, University Clinic for Fertility, Horsens, Denmark; Section of Biostatistics, Department of Public Health, University of Copenhagen, Copenhagen, Denmark; Fertility Clinic, Department of Gynaecology, Fertility and Obstetrics, Copenhagen University Hospital—Rigshospitalet, Copenhagen, Denmark; Fertility Clinic, Department of Gynaecology, Fertility and Obstetrics, Copenhagen University Hospital—Rigshospitalet, Copenhagen, Denmark; Department of Clinical Medicine, University of Copenhagen, Copenhagen, Denmark; Fertility Clinic, Department of Gynaecology, Fertility and Obstetrics, Copenhagen University Hospital—Rigshospitalet, Copenhagen, Denmark; Department of Clinical Medicine, University of Copenhagen, Copenhagen, Denmark

**Keywords:** frozen embryo transfer, natural cycle, modified natural cycle, progesterone, luteal phase support, timing, FET, mNC-FET, IVF, P4

## Abstract

**STUDY QUESTION:**

Can the use of progesterone luteal phase support (LPS) and timing of blastocyst transfer, at 6 versus 7 days following hCG-trigger, improve the live birth rate (LBR) in modified natural cycle (mNC) frozen-thawed embryo transfer (FET)?

**SUMMARY ANSWER:**

Use of LPS and advanced timing of blastocyst transfer did not significantly improve the LBR in mNC-FET.

**WHAT IS KNOWN ALREADY:**

Transfer of a frozen-thawed blastocyst in the mNC protocol is routinely conducted 7 days after the ovulation trigger, and progesterone LPS is commonly used despite limited evidence.

**STUDY DESIGN, SIZE, DURATION:**

This multicentre, single-blinded, randomized controlled superiority trial investigated the effect of LPS and timing of blastocyst transfer on live birth rates following mNC-FET conducted from January 2019 to February 2024. Using an online randomization programme, patients were randomized 1:1:1:1 to: (A) transfer day 6 following ovulation trigger (Day 0) with LPS; (B) transfer day 7 with LPS; (C) transfer day 6 without LPS; (D) transfer day 7 without LPS. Use of LPS was masked from treating clinicians. The sample size calculation required 604 women to participate to detect an increase in live birth rate from 21% in control groups (LPS: C + D, timing: B + D) to 31% in intervention groups (LPS: A + B, timing: A + C). In total, 679 women were enrolled, and 610 women were randomized. Ultimately, 602 women (A: n = 151; B: n = 150; C: n = 149; D: n = 152) were included in the per-transfer analyses.

**PARTICIPANTS/MATERIALS, SETTING, METHODS:**

Participants were women aged 18–41 years undergoing mNC-FET with a single autologous good-quality blastocyst, from eight public fertility clinics at tertiary care centres across Denmark.

**MAIN RESULTS AND THE ROLE OF CHANCE:**

Our study found that use of LPS did not significantly affect the LBR compared with no LPS: 34.9% (105/301) versus 31.9% (96/301) (adjusted risk difference (aRD) 3.18, 95% CI: −4.13 to 10.49; *P* = 0.39). Comparing blastocyst transfer on Day 6 versus Day 7, there was again no significant difference in LBR: 33.8% (99/300) versus 33.0% (99/302) (aRD −0.62, 95% CI: −7.99 to 6.75; *P* = 0.87).

**LIMITATIONS, REASONS FOR CAUTION:**

The sample size calculation was based on LBRs of studies using transfer of slow-frozen and cleavage stage embryos, but today’s LBRs are higher. Consequently, the sample size might not have been sufficient to detect discrete differences in reproductive outcomes under present conditions. Further, despite using liberal inclusion criteria, most women participating were young (≤35 years) and lean, and had a good quality embryo available for transfer, giving them an *a priori* good treatment prognosis. Hence, our findings might not apply to all women undergoing mNC-FET.

**WIDER IMPLICATIONS OF THE FINDINGS:**

This RCT demonstrates that supplementary progesterone likely does not improve live birth rates, and that flexibility in scheduling blastocyst transfers is possible for most women undergoing mNC-FET following their first to third IVF/ICSI treatment. Our results pave the way for a less restricted approach to FET, hence reducing patient burden and costs without compromising success rates.

**STUDY FUNDING/COMPETING INTEREST(s):**

Study funding was from Rigshospitalet’s Research Foundation and Gedeon Richter (forward grant, FORWARD2018_5). The funders of the study had no role in study design, data collection, data analysis, data interpretation, writing of this report, or the decision to submit the article for publication. M.S. reports an unrestricted research grant paid to the institution from Gedeon Richter to fund present work, a travel grant from Gedeon Richter paid to the institution, and personal honoraria for lectures from Gedeon Richter and IBSA. L.P. reports payments to the institution for travel from IBSA, Ferring Pharmaceuticals, and Merck, and payments to the institution from Ferring Pharmaceuticals for participation on a data safety monitoring board or advisory board. N.l.C.F. reports research grants paid to the institution from Gedeon Richter, Merck, and Cryos, personal consulting fee from Merck, personal honoraria for lectures from Merck, and travel grants paid to the institution from Merck, Ferring Pharmaceuticals, IBSA, and Gedeon Richter. Additionally, N.l.C.F. is a chair in the steering committee for the guideline groups for the Danish Fertility Society (unpaid). A.K. reports personal honoraria for lectures from Organon and Merck and travel grants paid to the institution from Merck and Ferring Pharmaceuticals. B.O.-M. reports travel grants paid to the institution by Gedeon Richter and personal fees for participation on a data safety monitoring board or advisory board from Ferring Pharmaceuticals. P.H. reports research grants paid to the institution from Merck and Gedeon Richter and personal honoraria for lectures from Merck, IBSA, and Gedeon Richter. E.C.L.L. reports personal honoraria for lectures from Pfizer and Gedeon Richter, travel grants from Merck and Gedeon Richter, and payment to the institution for participation on a data safety monitoring board or advisory board from Astellas Pharma. K.L. reports a research grant paid to the institution from Gedeon Richter, personal consulting fees from Ferring Pharmaceuticals, personal honorarium for a lecture from Ferring Pharmaceuticals, and personal travel grants from Ferring Pharmaceuticals and Merck. A.P. reports research grants from Gedeon Richter, Ferring Pharmaceuticals, and Merck, personal consulting fees from Gedeon Richter, Ferring Pharmaceuticals, and Merck, personal honoraria for lectures from Gedeon Richter, Ferring Pharmaceuticals, Merck, and Abbott, and a travel grant from Gedeon Richter. All other authors declare no competing interests.

**TRIAL REGISTRATION NUMBER:**

The trial was registered with ClinicalTrials.gov (NCT03795220) and EudraCT (2018-002207-34) and is completed.

**TRIAL REGISTRATION DATE:**

The study was registered at clinicaltrials.gov on 4 December 2018.

**DATE OF FIRST PATIENT’S ENROLMENT:**

The first patient was enrolled on 6 January 2019.

WHAT DOES THIS MEAN FOR PATIENTS? This study investigates whether using vaginal progesterone suppositories and changing the day of embryo transfer in modified natural cycle (mNC) protocols for frozen embryo transfer (FET) can increase a woman’s chance of having a baby. Despite numerous FET treatments being performed each year, the optimal timing of embryo transfer has not been established, and furthermore, progesterone is commonly used despite limited proof of its effect. This research trial was conducted at multiple assisted reproduction centres and included random groups of women with similar fertility characteristics. We found that use of vaginal progesterone in the mNC protocol for FET did not increase the chances of having a baby, and that having embryo transfer on either Day 6 or Day 7 after ovulation trigger resulted in equally good treatment results. Our results open the door to fertility treatment with less medication and more flexibility in planning.

## Introduction

The global landscape of ART is rapidly evolving, with frozen embryo transfers (FETs) now comprising close to half of all ART cycles performed worldwide (Adamson *et al.*, 2025). This development underscores an important need to identify the most efficient FET protocol. Despite there being no consensus on the best FET protocol in terms of reproductive outcomes, the natural cycle (NC) protocols are gaining popularity. Recent studies have shown that the risk of complications related to hypertensive disorders of pregnancy is lower in NC-FET, including the modified (m) NC protocol, compared to the historically preferred hormone replacement therapy (HRT) protocol, encouraging clinicians to consider a more natural approach (von Versen-Höynck *et al.*, 2019; Zaat *et al.*, 2023).

In all FET protocols, ensuring a favourable endocrine milieu during implantation and early pregnancy is pivotal. Although women undergoing NC-FET ovulate and produce a corpus luteum (CL) and endogenous progesterone, additional luteal phase support (LPS) with progesterone is routinely used. The rationale of using progesterone LPS is to stabilize the endometrium, increase the chance of implantation, support early pregnancy development, and potentially rescue cycles with an inadequate endogenous progesterone production. For that reason, we hypothesized that use of progesterone LPS in the mNC protocol for FET would improve the overall live birth rate (LBR).

Strongly associated to treatment success in FET is synchronicity between the developmental stage of the embryo and the endometrium (Hertig *et al.*, 1956; Psychoyos, 1974; Wilcox *et al.*, 1999). In the mNC protocol, ultrasonic assessment of follicular growth is used to decide the optimal time to trigger ovulation and to perform the blastocyst transfer; when a mature follicle (≥17 mm) is detected, the ovulation trigger is administered on the same day, and a blastocyst transfer is planned. However, relying on findings from ultrasound alone when deciding on the optimal time to trigger ovulation can be challenging. While the size of the leading follicle, the oestradiol level, and the endogenous LH surge are connected, some women may be on the plateau, or even at the descent of their LH surge, by the time their leading follicle is mature. Consequently, some women have entered the luteal phase when the ovulation trigger is given, putting them at risk of embryo–endometrial asynchrony. Further, cell loss from the blastocyst during the vitrification-warming procedure could hypothetically cause a developmental delay, corresponding to an earlier developmental stage. Thus, we hypothesized that advancing the day of blastocyst transfer by one day, i.e. to Day 6 (novel approach) instead of Day 7 (routine) following the ovulation trigger, would minimize the risk of embryo–endometrial asynchrony and lead to a superior LBR. Hence, in this randomized controlled trial (RCT), we compared LBRs in patients undergoing mNC-FET with and without the use of progesterone LPS and with blastocyst transfer on Day 6 versus Day 7 following the ovulation trigger,

## Materials and methods

### Study design

The study was a multicentre, single-blinded, randomized controlled trial conducted at eight public fertility clinics across four Danish regions between January 2019 and March 2024. Patients were continuously included during the above period, except during the COVID-19 pandemic of Spring 2020. Active recruitment ended when 604 patients were randomized to the trial. Patient follow-up was concluded in December 2024. Data were collected by allocated staff at the participating sites. Data management and analysis were conducted at the primary research centre: Copenhagen University Hospital Rigshospitalet, Denmark. An independent statistician overlooked all statistical analyses. The protocol has previously been published (Saupstad *et al.*, 2019).

### Ethical approval

The study received initial approval by The Capital Region of Denmark Centre for Ethics, H-18025839, 6 September 2018, The Capital Region of Denmark, Research and Innovation, Legal Department (data storage) P-2019-670, 6 November 2019, and the Danish Medicines Agency, 63569, 8 August 2018.

### Participants

Women eligible for study participation were 18–41 years old with regular menstrual cycles (23–35 days), scheduled to undergo single, autologous blastocyst transfer in an mNC endometrial preparation protocol following their first, second, or third oocyte retrieval at one of the participating clinics. Only women with a good-quality blastocyst (expansion score 3–6 on vitrification day 5 or expansion score 4–6 on vitrification day 6 after oocyte retrieval, inner cell mass/trophectoderm score A or B according to the Gardner scoring system (Gardner and Schoolcraft, 1999)) were eligible for participation. Exclusion criteria were: previous study participation, recipients of oocyte donation, preimplantation genetic testing (PGT) for heritable diseases, uterine malformations, severe uterine pathology (polyps or submucosal myomas), blastocyst conceived with sperm from testicular sperm aspiration, ongoing HIV or Hepatitis B or C infection, previous treatment for suspected luteal phase insufficiency, and contraindications to treatment with progesterone LPS provided by the manufacturer (Lutinus, Ferring Pharmaceuticals, Kastrup, Denmark and Cyclogest, Gedeon Ricter Nordics AB, Stockholm, Sweden). PGT for aneuploidy was not performed and is restricted by law in Denmark.

Patients eligible for study participation were invited to participate through oral and written patient information provided by research staff. Patients accepting study participation were booked for a baseline visit on cycle days 2–5 to confirm eligibility. All women enrolled in the trial provided written informed consent for trial participation and storage of blood samples in a biobank. Both participating women and partners provided written consent for follow-up on children conceived in the trial.

### Randomization and masking

Women included in the RCT were monitored with continuous measurements of follicular growth and assessment of the endometrium by transvaginal ultrasound (TVUS) from the mid-follicular phase (cycle days 8–12, depending on cycle length), until the dominant follicle reached 15–17 mm. Ultrasound scans were performed no more than 3 days apart, and often daily as the leading follicle developed. Whenever feasible, women were followed until the dominant follicle reached ≥17 mm and given the ovulation trigger on the same evening (Day 0). If the clinic or patient were unable to accommodate daily scans in the late follicular phase, women with a follicle of 15–16 mm were instructed to administer the ovulation trigger the following evening (Day 0). On the day of hCG trigger, or the day before, women were allocated to one of the four study groups (1:1:1:1) by randomization: (A) progesterone LPS and blastocyst transfer on Day 6 following ovulation trigger, (B) LPS and blastocyst transfer on Day 7, (C) no LPS and blastocyst transfer on Day 6, and (D) no LPS and blastocyst transfer on Day 7. Randomization was conducted by a research staff member, using a computerized programme running a minimization algorithm (dOxos Clinical Data Services AB, Gothenburg, Sweden). To ensure equal distribution of traits associated with treatment outcomes, the following variables were balanced by the randomization algorithm: study site (1 to 8), age (years), number of IVF/ICSI treatments (1–3), number of FET treatments (1, 2, or ≥3), and blastocyst expansion grade (Gardner expansion score 3, 4, and 5/6). Treating clinicians were blinded to the use of LPS, but patients and research staff members were not.

### Procedures

Before the FET cycle, ovarian stimulation (OS) and oocyte retrieval were conducted according to either the gonadotropin-releasing hormone (GnRH) antagonist or agonist protocol at the local trial site. Blastocysts were cultured for 5 or 6 days before assessment of blastocyst score and vitrification. On the day of transfer, blastocysts were warmed in the morning and transferred at midday. FET was conducted in the second or a subsequent cycle following OS and oocyte retrieval at all study sites.

During the FET cycle, when the dominant follicle reached ≥17 mm, the hCG trigger (250 µg human chorionic gonadotropin, Ovitrelle, Merck A/S, Søborg, Denmark) was administered at 10 pm the same evening. Women randomized to LPS (study groups A and B) were instructed by a research staff member to administer vaginal suppositories (100 mg * 3/daily, Lutinus, Ferring Pharmaceuticals or 400 mg * 2/daily Cyclogest, Gedeon Richter Nordics AB.) starting the morning of Day 4 following the ovulation trigger. All study medication was handed to the patients free of charge. Individual medicine accounts were kept by the clinic, and complete medicine accounts were kept by the primary study site. Patients were instructed to inform the clinic about deviations in medicine administration, adverse events (sickness, admittance to hospital, etc.), and initiation of concomitant prescription medication until gestational age (GA) 8 + 0.

On Day 6 (study groups A and C) or 7 (study groups B and D) following the hCG trigger, single blastocyst transfer was planned according to the allocated study group. In case the appointed blastocyst did not survive thawing, another blastocyst was thawed if available. If not, the treatment cycle was cancelled. Patients randomized to LPS (study groups A and B) were asked to postpone the morning dose of progesterone until after blastocyst transfer, to avoid unblinding of the treating doctor.

Following blastocyst transfer, patients were scheduled for pregnancy testing (serum (s) hCG) 16 days after the administration of the ovulation trigger. An s-hCG level of ≥5 IU/l was considered a positive test. Patients with an s-hCG level ≥50 IU/l were invited to a pregnancy scan during gestational week 8, while an s-hCG level between 5 and 49 IU/l necessitated repeated s-hCG testing until a sufficient rise or a decline <5 IU/l was detected. An s-hCG level <5 IU/l was considered a negative test, and patients were discontinued from the trial. If pregnant, patients receiving LPS (study groups A and B) continued treatment until GA 8 + 0. Follow-up on pregnancy outcome, obstetric complications, and perinatal outcomes was conducted on all patients with an ultrasound-verified live pregnancy past GA 7 + 0.

### Outcomes

The primary outcome was LBR per randomized patient. Key secondary outcomes were pregnancy rate (PR) defined as an s-hCG ≥5 IU/l 16 days after administration of the ovulation trigger, and rate of clinical pregnancies (CPR) defined here as an intrauterine pregnancy with a foetal heartbeat visualized by TVUS at GA 7–8 weeks. Other secondary outcomes included the total pregnancy loss rate (PLR), including all cases of pregnancy loss following a positive s-hCG, i.e. early miscarriage (pregnancy loss following a positive pregnancy test, before verification of a clinical pregnancy), clinical miscarriage (pregnancy loss after verification of a clinical pregnancy, before GA 22 + 0), ectopic pregnancy, and induced abortion. For singleton pregnancies that resulted in delivery after GA 22 + 0, obstetric complications, including hypertensive disorders of pregnancy, gestational diabetes, postpartum haemorrhage, and caesarean section, were reported. Perinatal outcomes, including preterm birth, birth weight, congenital malformations, and perinatal mortality (foetal and neonatal death from GA 22 + 0 to 7 days after birth) were recorded.

### Statistical analysis

The RCT was designed as a superiority trial, using a 2 × 2 factorial design, assessing if mNC-FET with LPS is superior to mNC-FET without LPS (study groups A + B vs C + D) and if mNC-FET with blastocyst transfer on Day 6 following ovulation trigger is superior to blastocyst transfer on Day 7 following ovulation trigger (study groups A + C vs B + D). The two interventions were considered independent of each other. Sample size calculation required 604 women to participate, equalling 151 patients in each study group, to detect an increase in live birth rate from 21% in groups C + D to 31% in groups A + B (and similarly in groups B + D to A + C) with 80% power and a 0.05% level of significance. A 10% difference in live birth rate was considered clinically significant. After initiation of the study, the number of participants needed to meet the required sample size was elevated to compensate for drop-outs (expected at 10–20%).

Baseline characteristics were reported for the per-transfer (PT) population (all randomized, undergoing embryo transfer). Primary and secondary reproductive outcomes were reported for the PT, intention-to-treat (ITT) (all randomized), per-protocol (PP) (all randomized, receiving allocated treatment), and as-treated (AT) (all randomized, grouped according to the treatment received) populations. Obstetric complications of pregnancy and perinatal outcomes were reported per singleton delivery in the PT, PP, and AT populations. Patients withdrawing from the trial at any stage were removed from all analyses. In case of missing data, it is disseminated in the associated tables.

For comparisons of primary and secondary reproductive outcomes, we calculated adjusted risk differences (aRD) using logistic regression followed by direct standardization, while adjusting for the following covariates: use of LPS (yes/no), blastocyst transfer (Day 6/Day 7), study site (1–8), age (20–29, 30–34, ≥35), number of oocyte retrieval cycles (1, 2, 3), number of FET treatments (1, 2, 3 or more), expansion grade of best rated blastocyst (grade 3, 4, and 5/6) in addition to parity (yes/no), and day of blastocyst vitrification (Day 5/Day 6). For comparisons of binary obstetric complications and perinatal outcomes, we used the Chi-squared test (n events >10) or Fisher’s exact test (n events ≤10) due to the rarity of these outcomes. A *t*-test was used to calculate the *P*-value for continuous data. A two-tailed *P*-value of <0.05 was considered statistically significant. All analyses were made using R Studio, version 2023.06.0, build 421 and the risks-package (Stopsack and Gerke, 2023). The full statistical analysis plan is found in Supplementary File S1.

The trial was monitored by the local Good Clinical Practice units of the Capital Region of Denmark, Central Denmark Region, and the North Denmark Region, ensuring proper conduct of research. The trial was registered with ClinicalTrials.gov on 4 December 2018 (NCT03795220) and EudraCT 07 June 2018 (2018-002207-34).

### Patient and public involvement

Development of this research project, implementation, recruitment of patients, and interpretation of results were conducted without patient involvement; however, experiences of the first patients included in the study encouraged us to include a survey on self-reported health and wellbeing (Colombo *et al.*, 2023).

## Results

**Figure 1.**
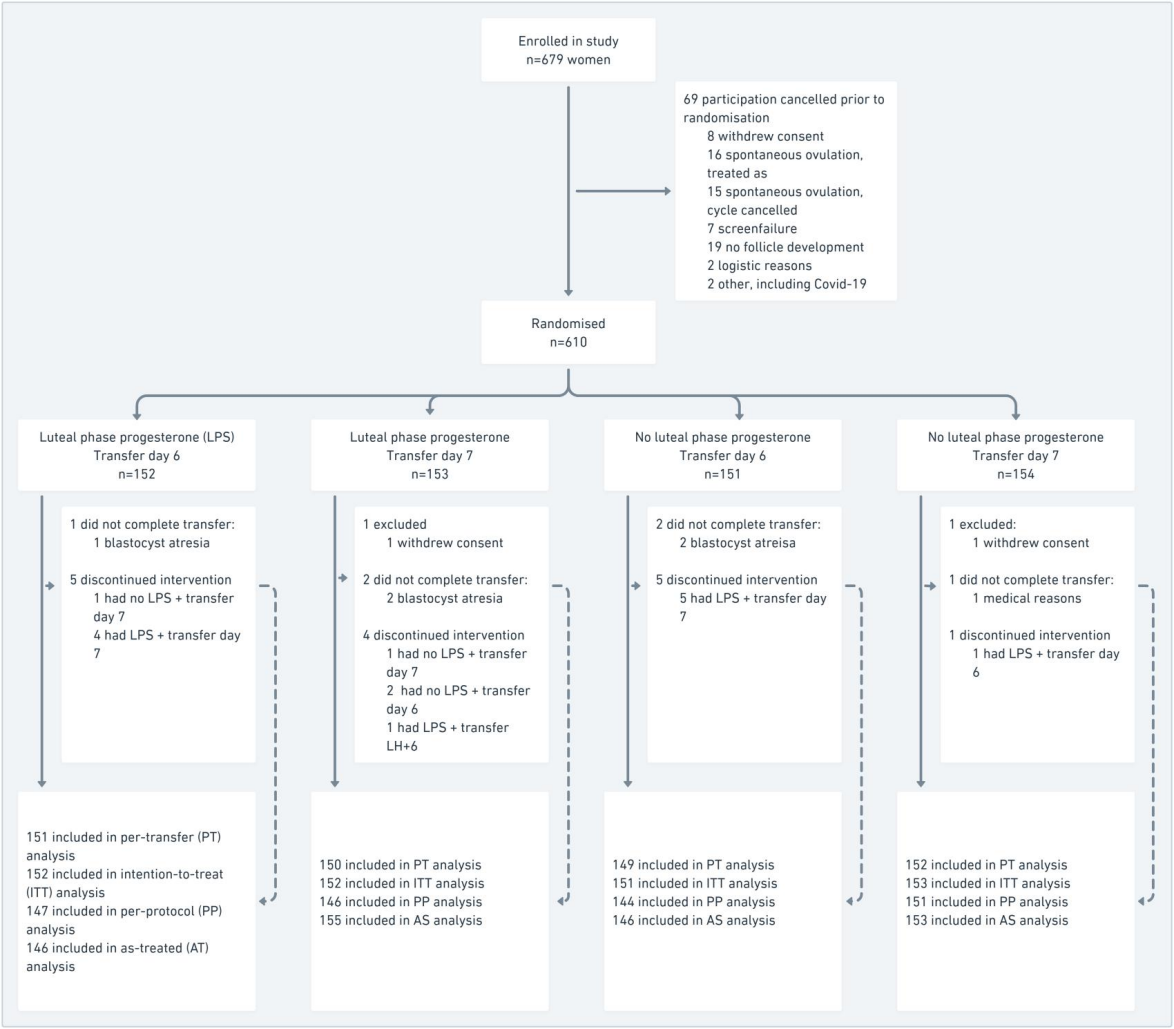
**Inclusion flowchart.** Per-transfer analyses included women randomized, undergoing embryo transfer. Intention-to-treat analyses included all women randomized. Per-protocol analyses included women randomized, undergoing allocated treatment. As-treated analyses included women randomized, grouped according to treatment received. LPS, luteal phase support (progesterone); PT, per-transfer; ITT, intention-to-treat; PP, per-protocol; AS, as-treated.

From January 2019 to February 2024, 679 women were included in the trial. Of these, 610 women (89.8%) were randomly assigned to one of the four study groups (A: n = 152, 24.9%, B: n = 153, 25.1%, C: n = 151, 24.8%, D: n = 154, 25.3%), while 69 women were not randomized, mostly due to spontaneous ovulation (n = 31) or lack of follicular development (n = 19). Of the 610 women randomized, two women withdrew consent and six women failed to undergo transfer (A: n = 1, 0.7%, B: n = 3, 2.0%, C: n = 2, 1.3%, D: n = 2, 1.3%). Ultimately, 602 women were included in the per-transfer analysis (A: n = 151, B: n = 150, C: n = 149, D: n = 152). Follow-up of study participants was concluded in December 2024. A CONSORT flowchart is depicted in Fig. 1.

The median (IQR) age of participating women was 33.0 (30–36) years. Among the study participants, 86.2% (519/602) had a male partner, 9.8% (24/602) were single women, and 4.0% (59/602) were living in same-sex couples. The majority were of Caucasian ethnicity (92.0%, 554/602), reflecting the general composition of the Danish population. Most participants were enrolled following their first oocyte retrieval cycle (70.6%, 425/602) and were participating in their first FET treatment (62.3%, 375/602). In all study groups, approximately 3 in 4 women were nulliparous. See Tables 1 and 2 for full baseline and treatment characteristics.

**Table 1.** Demographic, baseline, and cycle characteristics in the per-transfer population.

	Luteal phase progesterone	Luteal phase progesterone	No luteal phase progesterone	No luteal phase progesterone
	Transfer Day 6^a^	Transfer Day 7^a^	Transfer Day 6^a^	Transfer Day 7^a^
	(n = 151)	(n = 150)	(n = 149)	(n = 152)
**Age, years**	32 (30–37)	33 (29–36)	33 (30–36)	33 (30–37)
**Age categories**				
Age ≥35 years	58 (38.4)	56 (37.3)	55 (36.9)	64 (42.1)
Age ≥37 years	40 (26.5)	33 (22.0)	33 (21.1)	39 (25.7)
**Ethnicity**				
Caucasian	142 (94.0)	132 (88.0)	137 (91.9)	143 (94.1)
Other	9 (6.0)	18 (12.0)	12 (8.1)	9 (5.9)
**Civil status**				
Heterosexual couple	122 (80.8)	133 (88.7)	131 (87.9)	133 (87.5)
Homosexual couple	9 (6.0)	5 (3.3)	4 (2.7)	6 (4.0)
Solo	20 (13.2)	12 (8.0)	14 (9.4)	13 (8.6)
**Body mass index, kg/m^2^, median (IQR)**	23.5 (21.6–25.8)	23.7 (21.0–26.8)	23.5 (21.0–27.1)	22.7 (20.6–26.4)
Data missing	1 (0.7)	1 (0.7)	0	1 (0.7)
**Smoking, daily**	2 (1.3)	2 (1.3)	6 (4.0)	6 (4.0)
Data missing	1 (0.7)	0	0	1 (0.7)
**Menstrual cycle length, days**	28 (28–30)	28 (28–30)	29 (28–30)	28 (27–29)
**Duration of infertility,^b^ years, median (IQR)**	2.5 (2.0–3.3)	2.0 (1.8–3.0)	2.8 (2.1–3.7)	2.5 (2.0–3.0)
Data missing	1 (1.9)	4 (7.8)	0	3 (4.8)
**AMH,^c^ pmol/l, median (IQR)**	17 (10–25)	17 (9–28)	21 (12–30)	17 (11–25)
Data missing	13 (8.6)	12 (8.0)	12 (8.1)	13 (8.6)
**Antral follicle count**	18 (13–25)	16 (12–25)	19 (12–27)	19 (12–24)
Data missing	1 (0.7)	4 (2.7)	2 (1.3)	4 (2.6)
**Cause of infertility** ^d^				
Female factor	22 (14.6)	21 (14.0)	28 (18.8)	27 (17.8)
Male factor	69 (45.7)	68 (45.3)	79 (53.0)	79 (52.0)
Unexplained	39 (25.8)	52 (34.7)	42 (28.2)	39 (25.7)
Other	30 (19.9)	19 (12.7)	19 (12.8)	23 (15.1)
**Fertility treatment history**				
First IVF/ICSI	105 (69.5)	106 (70.7)	108 (72.5)	106 (69.7)
Second IVF/ICSI	34 (22.5)	32 (21.3)	24 (16.1)	34 (22.4)
Third IVF/ICSI	12 (8.0)	12 (8.0)	17 (11.4)	12 (7.9)
First FET	92 (60.9)	91 (60.7)	97 (65.1)	95 (62.5)
Second FET	36 (23.8)	37 (24.7)	34 (22.8)	33 (21.7)
Third or more FET	23 (15.2)	22 (14.7)	18 (12.1)	24 (15.8)
**Reproductive history**				
Previous birth	38 (25.2)	39 (26.0)	42 (28.2)	34 (22.4)
Previous pregnancy	86 (57.0)	86 (57.3)	100 (67.1)	80 (52.6)

a Data are n (%) unless otherwise stated. Data are presented as intention-to-treat, including all women randomized, not including women who withdrew consent after randomization (n = 2). No significant differences between groups (*P* < 0.05) were present in any of the baseline characteristics.

b Data only available for women in a heterosexual relationship without a previous pregnancy.

c Several patients have more than one registered cause of infertility. The category “Other” includes women without a partner and women with a female partner.

d AMH was not measured at study site 8.

IQR, interquartile range.

**Table 2.** Day of ovulation trigger, blastocyst, and blastocyst transfer characteristics in the per-transfer population.

	Luteal phase progesterone	Luteal phase progesterone	No luteal phase progesterone	No luteal phase progesterone
	Transfer Day 6^a^	Transfer Day 7^a^	Transfer Day 6^a^	Transfer Day 7^a^
	(n = 151)	(n = 150)	(n = 149)	(n = 152)
**Trigger day characteristics**				
**Cycle day of ovulation trigger (days), median (IQR)**	12 (11–14)	12 (11–14)	13 (11–15)	12 (11–15)
**Size of leading follicle at trigger (mm),^b^ median (IQR)**	17.0 (17–18)	17.5 (17–19)	17.0 (17–18)	17.5 (17–18)
Data missing	0	0	0	1 (0.9)
**Endometrial thickness (mm), median (IQR)**	8.1 (7.1–9.5)	8.2 (7.2–9.3)	8.2 (7.3–10.0)	8.1 (7.4–10.0)
Data missing	1 (0.9)	0	0	0
**Blastocyst characteristics ^c^**				
**Method of fertilization**				
IVF	74 (49.0)	75 (50.0)	68 (45.6)	66 (43.4)
ICSI	77 (51.0)	75 (50.0)	81 (54.4)	86 (56.6)
**Blastocyst Gardner score**				
5/6 AA, AB, BA, BB	41 (27.2)	39 (26.0)	37 (24.8)	39 (25.7)
4 AA, AB, BA, BB	77 (51.0)	79 (52.7)	79 (53.0)	80 (52.6)
3 AA, AB, BA, BB	33 (21.9)	32 (21.3)	33 (22.1)	33 (21.7)
**Day of blastocyst vitrification**				
Day 5	107 (70.9)	112 (74.7)	117 (78.5)	112 (73.7)
Day 6	44 (29.1)	38 (25.3)	32 (21.5)	40 (26.3)

a Data are n (%) unless otherwise stated. Trigger day and blastocyst characteristics data are presented as per-transfer, including all women undergoing embryo transfer. No significant differences between groups (*P* < 0.05) were present in any of the baseline characteristics.

b Data shown for patients that underwent transvaginal ultrasound on the day of ovulation trigger: *N* = 447. Group A, n = 113. Group B, n = 117. Group C, n = 106. Group D, n = 107. The remaining patients were scanned the day prior to the ovulation trigger, subject to clinic opening hours, etc.

c Describes blastocyst rated best at time of inclusion. Three patients were included, with a blastocyst not fitting the inclusion criteria. n = 33 patients were transferred with a blastocyst other than the one rated best at inclusion. Only 10 patients had a blastocyst transferred that did not meet the inclusion criteria.

### Luteal phase support

Comparing women with and without LPS, live birth occurred in 34.9% (105/301) versus 31.9% (96/301) of women (aRD 3.18, 95% CI: −4.13 to 10.49; *P* = 0.39) (Table 3), thus the superiority of LPS was not confirmed. For the secondary outcomes, we found no significant difference in PRs, CPRs, and PLRs between the two groups, with and without LPS. Pregnancy occurred in 50.8% (153/301) of women receiving LPS versus 49.8% (150/301) of women not receiving LPS (aRD 1.36, 95% CI: −6.35 to 9.08; *P* = 0.73) (Table 3). Clinical pregnancy with foetal heartbeat occurred in 38.2% (115/301) of women receiving LPS versus 33.2% (100/301) of women not receiving LPS (aRD 5.29, 95% CI: −2.16 to 12.75; *P* = 0.16) (Table 3). Lastly, pregnancy loss occurred in 15.9% (48/301) of women allocated to LPS versus in 17.9% (54/301) without LPS (aRD −1.85, 95% CI: −7.76 to 4.05; *P* = 0.54) (Table 3); of these 12.0% (36/301) versus 15.9% (48/301) were early miscarriages (*P* = 0.20) (Supplementary Table S1) in the LPS versus no LPS groups, while 2.7% (8/301) versus 1.0% (3/301) (*P* = 0.22) (Supplementary Table S1) were clinical miscarriages in the LPS versus no LPS groups. ITT, PP, and AT analyses confirmed the above results. Lastly, no statistically significant differences were found in obstetric complications and perinatal outcomes comparing the LPS versus the no LPS groups (Table 4).

**Table 3.** Reproductive outcomes for women randomized to luteal phase progesterone support versus no luteal phase progesterone support.

	Luteal phase progesterone Groups A + B, n (%)	No luteal phase progesterone Groups C + D, n (%)	Unadjusted risk difference, % (95% CI)	Unadjusted *P*-value*	Adjusted risk difference^a^, % (95% CI)	Adjusted *P*-value*
**Per-transfer^b^**						
Live birth	105 (34.9)	96 (31.9)	2.99 (4.54–10.52)	0.44	3.18 (−4.13–10.49)	0.39
Clinical pregnancy with fetal heartbeat	115 (38.2)	100 (33.2)	4.98 (−2.66–12.63)	0.20	5.29 (−2.16–12.75)	0.16
Pregnancy	153 (50.8)	150 (49.8)	1.00 (−6.99–8.98)	0.81	1.36 (−6.35–9.08)	0.73
Pregnancy loss	48 (15.9)	54 (17.9)	−1.99 (−7.99–4.00)	0.51	−1.85 (−7.76–4.05)	0.54
**Intention-to-treat^c^**						
Live birth	105 (34.5)	96 (31.6)	2.96 (−4.51–10.43)	0.44	3.39 (−3.86–10.64)	0.36
Clinical pregnancy with fetal heartbeat	115 (37.8)	100 (32.9)	4.93 (−2.66–12.53)	0.20	5.48 (−1.92–12.88)	0.15
Pregnancy	153 (50.3)	150 (49.3)	1.00 (−6.96–8.93)	0.81	1.72 (−5.96–9.40)	0.66
Pregnancy loss	48 (15.8)	54 (17.8)	−1.97 (−7.91–3.96)	0.52	−1.73 (−7.58–4.12)	0.56
**Per-protocol^d^**						
Live birth	102 (34.9)	96 (32.5)	2.39 (−5.26–10.04)	0.54	2.66 (−4.79–10.10)	0.48
Clinical pregnancy with fetal heartbeat	112 (38.4)	100 (33.9)	4.46 (−3.31–12.22)	0.26	4.86 (−2.72–12.45)	0.21
Pregnancy	148 (50.7)	149 (50.5)	0.18 (−7.91–8.27)	0.97	0.46 (−7.37–8.28)	0.91
Pregnancy loss	46 (15.8)	53 (18.0)	−2.21 (−8.27–3.84)	0.47	−2.18 (−8.13–3.77)	0.47
**As-treated^e^**						
Live birth	103 (34.1)	98 (32.8)	1.33 (−6.21–8.87)	0.73	1.85 (−5.49–9.20)	0.62
Clinical pregnancy with fetal heartbeat	113 (37.4)	102 (34.1)	3.30 (−4.36–10.96)	0.40	4.07 (−3.41–11.56)	0.29
Pregnancy	152 (50.3)	151 (50.5)	−0.17 (−8.17–7.82)	0.97	0.36 (−7.40–8.11)	0.93
Pregnancy loss	49 (16.2)	53 (17.7)	−1.50 (−7.50–4.50)	0.62	−1.49 (−7.43–4.45)	0.62

a Adjusted risk differences were calculated using logistic regression, followed by direct standardization, adjusting for the following covariates: use of luteal phase support (LPS), time of blastocyst transfer, study site, age, number of oocyte pick-ups, number of frozen embryo transfers, expansion grade of best rated blastocyst, parity, and day of blastocyst vitrification.

b Per-transfer analyses: Included women randomized, undergoing embryo transfer, *N* = 602. Groups A + B, n = 301. Groups C + D, n = 301.

c Intention-to-treat analyses: Included women randomized, *N* = 608. Groups A + B, n = 304, Groups C + D, n = 304.

d Per-protocol analyses: Included women randomized, undergoing allocated treatment, *N* = 587. Groups A + B, n = 292. Groups C + D, n = 295.

e As-treated analyses: Included women randomized, grouped according to treatment received (a total of n = 6 women had LPS, although they should not have according to group allocation), *N* = 601. Groups A + B, n = 302. Groups C + D, n = 299.

* A *P*-value of <0.05 was considered statistically significant.

Definitions: Live birth is defined as the birth of a live child following 22 weeks of gestation. Clinical pregnancy with fetal heartbeat is defined as an ultrasound-verified intrauterine live pregnancy after 7 weeks of gestation. Pregnancy is defined as serum hCG ≥ 5IU/ml measured 16 days following the ovulation trigger.

CI, confidence interval.

**Table 4.** Obstetric and perinatal outcomes for singleton**^a^** livebirths from women randomized to luteal phase progesterone support versus no luteal phase progesterone support.

	Luteal phase progesterone	No luteal phase progesterone	*P*-value^*^^,c^
	Groups A + C^b^	Groups B + D^b^	
**Per-transfer ^d^**			
**Obstetric complications**			
Hypertensive disorders of pregnancy	14 (13.3)	8 (8.4)	0.38
Gestational diabetes	1 (1.0)	5 (5.3)	0.10
Postpartum haemorrhage	7 (6.7)	10 (10.5)	0.47
Caesarean section	29 (27.6)	20 (21.1)	0.36
**Perinatal outcomes**			
Preterm birth	6 (5.8)	4 (4.2)	0.75
Data missing	1 (0.5)	0	
Birthweight (g), mean (SD)	3515 (610)	3563 (569)	0.58
Data missing	4 (3.8)	2 (2.1)	
Low birth weight (<2500 g)	3 (3.0)	3 (3.2)	1.00
Very low birth weight (<1500 g)	1 (1.0)	0	1.00
High birth weight (>4000 g)	19 (18.8)	21 (22.6)	0.64
Very high birth weight (>4500 g)	3 (3.0)	5 (5.4)	0.48
Small for gestational age	7 (6.9)	4 (4.3)	0.63
Large for gestational age	2 (2.0)	3 (3.2)	0.67
Congenital malformation	6 (5.8)	2 (2.1)	0.28
Data missing	1 (0.9)	0	
Perinatal death	1 (1.0)	0	1.00
Data missing	1 (1.0)	0	
**Per-protocol ^e^**			
**Obstetric complications**			
Hypertensive disorders of pregnancy	14 (13.7)	8 (8.4)	0.34
Gestational diabetes	1 (1.0)	5 (5.3)	0.11
Postpartum haemorrhage	7 (6.9)	10 (10.5)	0.51
Caesarean section	27 (26.5)	20 (21.1)	0.47
**Perinatal outcomes**			
Preterm birth	6 (5.9)	4 (4.2)	0.75
Data missing	1 (1.0)	0	
Birthweight (g), mean (SD)	3501 (607)	3563 (569)	0.47
Data missing	3 (2.9)	2 (2.1)	
Low birth weight (<2500 g)	3 (3.0)	2 (2.2)	1.00
Very low birth weight (<1500 g)	1 (1.0)	0	1.00
High birth weight (>4000 g)	18 (18.2)	21 (22.6)	0.56
Very high birth weight (>4500 g)	3 (3.0)	5 (5.4)	0.49
Small for gestational age	7 (7.1)	4 (4.3)	0.61
Large for gestational age	1 (1.0)	3 (3.2)	0.36
Congenital malformation	6 (5.9)	2 (2.1)	0.28
Data missing	1 (1.0)	0	
Perinatal death	1 (1.0)	0	1.00
Data missing	1 (1.0)	0	
**As-treated ^f^**			
**Obstetric complications**			
Hypertensive disorders of pregnancy	14 (13.6)	8 (8.3)	0.33
Gestational diabetes	1 (1.0)	5 (5.2)	0.11
Postpartum haemorrhage	7 (6.8)	10 (10.3)	0.41
Caesarean section	27 (26.2)	22 (22.7)	0.68
**Perinatal outcomes**			
Preterm birth	6 (5.9)	4 (4.1)	0.75
Birthweight (g), mean (SD)	3501 (607)	3576 (571)	0.38
Data missing	4 (3.9)	2 (2.0)	
Low birth weight (<2500 g)	3 (3.0)	2 (2.1)	1.00
Very low birth weight (<1500 g)	1 (1.0)	0	1.00
High birth weight (>4000 g)	18 (18.2)	22 (23.2)	0.50
Very high birth weight (>4500 g)	3 (3.0)	5 (5.3)	0.49
Small for gestational age	7 (7.1)	4 (4.2)	0.58
Large for gestational age	1 (1.0)	4 (4.2)	0.21
Congenital malformation	6 (5.9)	2 (2.1)	0.28
Data missing	1 (1.0)	0	
Perinatal death	1 (1.0)	0	1.00
Data missing	1 (1.0)	0	

a Only one study participant gave birth to twins. Data on obstetric complications and perinatal outcomes are not reported but can be accessed by contacting the corresponding author.

b Data are n (%) unless otherwise stated.

c For comparisons of binary obstetric complications and perinatal outcomes, we used the Chi-squared test (n event >10) or Fisher’s exact test (n events ≤10) due to the rarity of these outcomes. A *t*-test was used to calculate the *P*-value for continuous data.

d Per-transfer analyses: Included women randomized, undergoing embryo transfer, who gave birth: *N* = 200. Groups A + B, n = 105. Groups C + D, n = 95.

e Per-protocol analyses: Included women randomized, undergoing allocated treatment, who gave birth: *N* = 197. Groups A + B, n = 102. Groups C + D, n = 95.

f As-treated analyses: Included women randomized, grouped according to treatment received (a total of n = 6 women had LPS although they should not have according to group allocation), who gave birth: *N* = 200. Groups A + B, n = 103. Groups C + D, n = 97.

* A *P*-value of <0.05 was considered statistically significant.

Definitions: Preterm birth was defined as a live birth before 37 weeks of gestation. Low birth weight was defined as a birth weight of less than 2500 g. Small and large for gestational age are defined as two standard deviation units below or above the expected birth weight. Perinatal mortality was defined as stillbirth or live birth followed by death within the first week following birth, from at least 22 completed gestational weeks.

CI, confidence interval.

### Timing of blastocyst transfer

Comparing blastocyst transfer Day 6 versus Day 7, live birth occurred in 33.8% (99/300) vs 33.0% (99/302) of women (aRD −0.62, 95% CI: 7.99–6.75; *P* = 0.87) (Table 5). Further, there were no statistically significant differences in PR, CPR, or PLR when comparing blastocyst transfer Day 6 versus Day 7 (Table 5). Statistically, the occurrence of gestational diabetes was higher (6.1%, 6/99, vs 0%, 0/101) in women undergoing blastocyst transfer on Day 6 versus Day 7, otherwise, no differences were found in the rate of obstetric complications and perinatal outcomes (Table 6).

**Table 5.** Reproductive outcomes for women randomized to blastocyst transfer Day 6 versus Day 7 following ovulation trigger.

	Transfer Day 6 Groups A + C, n (%)	Transfer Day 7 Groups B + D, n (%)	Unadjusted risk difference, % (95% CI)	Unadjusted *P*-value*	Adjusted risk difference^a^, % (95% CI)	Adjusted *P*-value*
**Per-transfer^b^**						
Live birth	99 (33.8)	99 (33.0)	−0.78 (−8.31–6.76)	0.84	−0.62 (−7.99–6.75)	0.87
Clinical pregnancy with fetal heartbeat	109 (36.3)	106 (35.1)	1.23 (−6.42–8.89)	0.75	1.38 (−6.13–8.89)	0.72
Pregnancy	152 (50.7)	151 (50.0)	0.67 (−7.32–8.66)	0.87	0.79 (−6.99–8.56)	0.84
Pregnancy loss	53 (17.7)	49 (16.2)	1.44 (−4.55–7.43)	0.64	1.29 (−4.68–7.25)	0.67
**Intention-to-treat^c^**						
Live birth	99 (32.7)	102 (33.4)	−0.77 (−8.25–6.71)	0.84	−0.70 (−8.02–6.63)	0.85
Clinical pregnancy with fetal heartbeat	109 (36.0)	106 (34.8)	1.22 (−6.28–8.82)	0.75	1.29 (−6.18–8.76)	0.74
Pregnancy	152 (50.2)	151 (49.5)	0.66 (−7.29–8.61)	0.87	0.62 (−7.12–8.37)	0.88
Pregnancy loss	53 (17.5)	49 (16.1)	1.43 (−4.51–7.37)	0.64	1.18 (−4.73–7.10)	0.70
**Per-protocol^d^**						
Live birth	97 (33.4)	101 (34.0)	−0.56 (−8.21–7.09)	0.87	−0.26 (−7.77–7.25)	0.95
Clinical pregnancy with fetal heartbeat	107 (37.0)	105 (35.4)	1.54 (−6.23–9.32)	0.70	1.74 (−5.92–9.40)	0.66
Pregnancy	147 (50.7)	150 (50.5)	0.19 (−7.91–8.27)	0.96	0.42 (−7.47–8.31)	0.92
Pregnancy loss	50 (17.2)	49 (16.5)	0.74 (−5.32–6.80)	0.81	0.51 (−5.50–6.53)	8.87
**As-treated^e^**						
Live birth	98 (33.4)	103 (33.4)	0.01 (−7.54–7.55)	1.00	0.43 (−6.98–7.83)	0.91
Clinical pregnancy with fetal heartbeat	108 (36.9)	107 (34.7)	2.12 (−5.55–9.79)	0.59	2.38 (−5.17–9.93)	0.54
Pregnancy	148 (50.5)	155 (50.3)	0.19 (−7.81–8.18)	0.96	0.63 (−7.18–8.43)	0.88
Pregnancy loss	50 (17.1)	52 (16.9)	0.18 (−5.82–6.19)	0.95	0.08 (−5.91–6.07)	0.98

a Adjusted risk differences were calculated using logistic regression, followed by direct standardization, adjusting for the following covariates: use of LPS, time of blastocyst transfer, study site, age, number of oocyte pick-ups, number of frozen embryo transfers, expansion grade of best rated blastocyst, parity, and day of blastocyst vitrification.

b Per-transfer analyses: Included women randomized, undergoing embryo transfer, *N* = 602. Groups A + C, n = 300. Groups B + C, n = 302.

c Intention-to-treat analyses: Included women randomized, *N* = 608. Groups A + C, n = 303. Groups B + C, n = 305.

d Per-protocol analyses: Included women randomized, undergoing allocated treatment, N = 587. Groups A + C, n = 290. Groups B + D, n = 297.

e As-treated analyses: Included women randomized, grouped according to treatment received, N = 601. Groups A + C, n = 293. Groups B + D, n = 308.

* A *P*-value of <0.05 was considered statistically significant.

Definitions: Live birth is defined as the birth of a live child following 22 weeks of gestation. Clinical pregnancy with fetal heartbeat is defined as an ultrasound-verified intrauterine live pregnancy after 7 weeks of gestation. Pregnancy is defined as serum hCG ≥ 5 IU/ml measured 16 days following ovulation trigger.

CI, confidence interval.

**Table 6.** Obstetric and perinatal outcomes for singleton**^a^** livebirths from women randomized to blastocyst transfer Day 6 versus Day 7 following ovulation trigger.

	Transfer Day 6	Transfer Day 7	*P*-value^*^^c^
	Groups A + C^b^	Groups B + D^b^	
**Per-transfer ^d^**			
**Obstetric complications**			
Hypertensive disorders of pregnancy	11 (11.1)	11 (10.9)	1.00
Gestational diabetes	6 (6.1)	0	0.01*
Postpartum haemorrhage	10 (10.1)	7 (6.9)	0.58
Caesarean section	26 (26.3)	23 (22.8)	0.68
**Perinatal outcomes**			
Preterm birth	3 (3.0)	7 (7.0)	0.33
Data missing	0	1 (1.0)	
Birthweight (g), mean (SD)	3539 (538)	3537 (641)	0.99
Data missing	1 (1.0)	5 (5.0)	
Low birth weight (<2500 g)	2 (2.0)	4 (4.1)	0.68
Very low birth weight (<1500 g)	0	1 (1.1)	0.50
High birth weight (>4000 g)	21 (21.4)	19 (19.8)	0.92
Very high birth weight (>4500 g)	3 (3.1)	5 (5.2)	0.50
Small for gestational age	6 (6.1)	5 (5.2)	1.00
Large for gestational age	2 (2.0)	3 (3.1)	0.68
Congenital malformation	4 (4.0)	4 (4.0)	1.00
Data missing	0	1 (1.0)	
Perinatal death	0	1	1.00
Data missing	0	1 (1.0)	
**Per-protocol ^e^**			
**Obstetric complications**			
Hypertensive disorders of pregnancy	11 (11.3)	1 (11.0)	1.00
Gestational diabetes	6 (6.2)	0	0.01*
Postpartum haemorrhage	10 (10.3)	7 (7.0)	0.57
Caesarean section	25 (25.8)	22 (22.0)	0.65
**Perinatal outcomes**			
Preterm birth	3 (3.1)	7 (7.1)	0.35
Data missing	0	1 (1.0)	
Birthweight (g), mean (SD)	3534 (539)	3528 (638)	0.94
Data missing	0	5 (5.0)	
Low birth weight (<2500 g)	2 (2.1)	3 (3.2)	0.68
Very low birth weight (<1500 g)	0	1 (1.0)	0.50
High birth weight (>4000 g)	21 (21.6)	18 (18.9)	0.78
Very high birth weight (>4500 g)	3 (3.1)	5 (5.3)	0.50
Small for gestational age	6 (6.2)	5 (5.3)	1.00
Large for gestational age	2 (2.1)	2 (2.1)	1.00
Congenital malformation	4 (4.1)	4 (4.0)	1.00
Data missing	0	1 (1.0)	
Perinatal death	0	1 (1.0)	1.00
Data missing	0	1 (1.0)	
**As-treated ^f^**			
**Obstetric complications**			
Hypertensive disorders of pregnancy	11 (11.2)	11 (10.8)	1.00
Gestational diabetes	6 (6.1)	0	0.01*
Postpartum haemorrhage	10 (10.2)	7 (6.9)	0.55
Caesarean section	26 (26.5)	23 (22.5)	0.62
**Perinatal outcomes**			
Preterm birth	3 (3.1)	7 (6.9)	0.33
Data missing	0	1 (1.0)	
Birthweight (g), mean (SD)	3543 (544)	3533 (636)	0.90
Data missing	0	6 (5.9)	
Low birth weight (<2500 g)	2 (2.0)	3 (3.1)	0.68
Very low birth weight (<1500 g)	0	1(1.0)	0.50
High birth weight (>4000 g)	22 (22.4)	18 (18.8)	0.65
Very high birth weight (>4500 g)	3 (3.1)	5 (5.2)	0.50
Small for gestational age	6 (6.1)	5 (5.2)	1.00
Large for gestational age	3 (3.1)	2 (2.1)	1.00
Congenital malformation	4 (4.1)	4 (4.0)	1.00
Data missing	0	1 (1.0)	
Perinatal death	0	1 (1.0)	1.00
Data missing	0	1 (1.0)	

a Only one study participant gave birth to twins. Data on obstetric complications and perinatal outcomes are not reported but can be accessed by contacting the corresponding author.

b Data are n (%) unless otherwise stated.

c For comparisons of binary obstetric complications and perinatal outcomes, we used the Chi-squared test (n event >10) or Fisher’s exact test (n events ≤10) due to the rarity of these outcomes. A *t*-test was used to calculate the *P*-value for continuous data.

d Per-transfer analyses: Included women randomized, undergoing embryo transfer, who gave birth: *N* = 200. Groups A + C, n = 99. Groups B + D, n = 101.

e Per-protocol analyses: Included women randomized, undergoing allocated treatment, who gave birth: *N* = 197. Groups A + C, n = 97. Groups B + D, n = 100.

f As-treated analyses: Included women randomized, grouped according to treatment received (a total of n = 6 women had LPS although they should not have according to group allocation), who gave birth: N = 200. Groups A + C, n = 98. Groups B + D, n = 102.

* A *P*-value of <0.05 was considered statistically significant.

Definitions: Preterm birth was defined as a live birth before 37 weeks of gestation. Low birth weight was defined as a birth weight of less than 2500 g. Small and large for gestational age are defined as two standard deviation units below or above the expected birth weight. Perinatal mortality was defined as stillbirth or live birth followed by death within the first week following birth, from at least 22 completed gestational weeks.

CI, confidence interval.

Supplementary Table S2 shows the occurrence of pregnancy loss, stratified by type, in the Day 6 versus Day 7 transfer groups.

Additionally, an overview of the primary and secondary reproductive outcomes for each of the four overall study groups can be found in Supplementary Table S3.

### Adherence to study interventions

#### Luteal phase support

Individual medicine accounts were kept by study staff at all sites and showed that no patients reported a delay in medicine administration or forgetting >2 vaginal suppositories. One patient (study group B) had to discontinue LPS due to illness, while 13 patients (study group A: n = 5, B: n = 2, C: n = 5, D: n = 1) randomized to blastocyst transfer on a clinic closing day had to discontinue the allocated intervention and instead had standard treatment, including routine use or not of LPS, according to local guidelines. In total, four patients did not use LPS that were randomized to use of LPS, while six patients had LPS that were not randomized to LPS (see Fig. 1). Patients who swapped treatment groups during the study were included in the PT and ITT analyses (in their designated group) and in the AT analyses (in the group in which they were treated), but not in the PP analyses.

#### Timing of blastocyst transfer

Due to logistic challenges (see above), 13 patients (study group A: n = 5, B: n = 2, C: n = 5, D: n = 1) randomized to blastocyst transfer on a clinic closing day had to discontinue the allocated intervention and instead had standard treatment with transfer the previous or following day, according to local guidelines. In total, nine patients had blastocyst transfer on Day 7, though they were designated to transfer on Day 6, while three patients had transfer on Day 6, though they were designated to transfer on Day 7.

### Harms

Congenital malformations were reported in 10 pregnancies: four in group A, three in group B, one in group C, and two in group D. One child in group B died two days following preterm birth at GA 29 + 0 due to preeclampsia and placental abruption. An overview of adverse obstetric and perinatal outcomes in the per-transfer population can be found in Supplementary Table S4.

## Discussion

In this RCT, we investigated two aspects of mNC-FET treatment: (i) the use of progesterone LPS and (ii) the timing of blastocyst transfer. Regarding the clinical effect of progesterone LPS, we found comparable LBRs, PRs, CPRs, and PLRs between groups using and not using LPS. As for the timing of blastocyst transfer, we did not see a statistically significant effect of performing blastocyst transfer on Day 6 compared to Day 7 on the LBRs, PRs, CPRs, or PLRs. Lastly, the use of LPS did not seem to impact the risk of obstetric complications and adverse perinatal outcomes.

The strengths of this study are the randomized controlled design, the multicentre set-up, and the substantial study population, making this the largest RCT investigating the effect of progesterone LPS and the timing of blastocyst transfer in mNC-FET to date. Further, we presented a low rate of protocol deviations, and no patient was lost to follow-up. Still, the trial has certain limitations. The most significant limitation is the possible lack of power. Our statistical assumptions were based on studies that included both slow-freeze and vitrification methods as well as cleavage-stage and blastocyst transfers. Given that current live birth rates are higher owing to exclusive use of vitrified blastocyst transfers, the final sample size in our study may not have been sufficient to detect small differences in reproductive outcomes under current clinical conditions. Further, no placebo was used in the no LPS group, and remnants of medication might have been present in the vagina at the time of embryo transfer in some patients. This may potentially have caused bias; still, we do not believe that remnants of progesterone or not in the vagina would have affected the way the clinicians performed the embryo transfer. Lastly, despite the liberal inclusion criteria, we acknowledge that most of the included patients were relatively young (≤35 years of age), lean, and had a good quality Day 5 blastocyst from their first IVF/ICSI cycle for transfer in their first FET cycle, giving our study population an *a priori* good treatment prognosis. Consequently, the results might not be applicable to all patients.

The use of progesterone LPS in mNC-FET did not significantly improve the treatment outcomes for our patients. While the absolute value of CPR was higher with the use of LPS, the risk difference in LBR between the groups was less pronounced and statistically insignificant, being lowest in the as-treated analysis with an aRD in LBR of <2%. Results from previous studies investigating the effect of LPS in mNC-FET are contradictory. Two smaller RCTs (*N* = 102 and *N* = 59, respectively) reported no benefit of using LPS on CPR or LBR (Eftekhar *et al.*, 2013; Horowitz *et al.*, 2021), while retrospective cohort studies reported inconsistent results. Two retrospective studies found no effect of LPS on ongoing pregnancy rate (*N* = 425) and LBR (*N* = 2216), and two studies found a positive effect of LPS on LBR (*N* = 228) and on CPR (*N* = 231) (Kyrou *et al.*, 2010; Kim *et al.*, 2014; Schwartz *et al.*, 2019; Ozmen and Ozer, 2025). Consequently, meta-analyses on the effect of LPS in mNC-FET, including sub-analyses on route and duration of progesterone administration, are much warranted.

Despite several studies indicating a beneficial effect of LPS in true (t) NC-FET conducted without an ovulation trigger (Bjuresten *et al.*, 2011; Gaggiotti-Marre *et al.*, 2020; Wånggren *et al.*, 2022), a definitive positive effect of LPS in mNC-FET has not been detected. The key might be the hCG trigger used in mNC-FET. A potential enhanced granulosa cell progesterone output elicited by hCG could strengthen the luteal phase (Bildik *et al.*, 2020), and thus, the need for exogenous progesterone in the early luteal phase of the mNC might be mitigated.

Our study is the first RCT to investigate the timing of blastocyst transfer and reproductive outcomes in mNC-FET, but a few retrospective studies have previously been published. In 2022, An *et al.* published a multicentre retrospective study on 1170 NC-FET cycles, finding that blastocyst transfers performed 7 days (160 ± 4–12 h) after ovulation trigger in mNC-FET resulted in a higher LBR compared to blastocyst transfers after 6 or 8 days (An *et al.*, 2022). While these results are in contrast with ours, our findings are in line with recent studies introducing new or modified NC/mNC-FET protocols supporting a notion of more temporal flexibility in the timing of blastocyst transfers (Weiss *et al.*, 2021; Alonso-Mayo *et al.*, 2023; Kornilov *et al.*, 2024; Mendes Godinho *et al.*, 2024). In 2023, Alonso-Mayo *et al.* proposed that a mature follicle might not be needed for treatment success in mNC-FET with progesterone LPS. The study assessed success rates based on the size of the dominant follicle at the time of the ovulation trigger, with endometrial thickness ≥7 mm and serum progesterone <1.5 ng/ml and found comparable reproductive outcomes across follicle diameters from 13 to 22 mm (Alonso-Mayo *et al.*, 2023). A year later, Mendes Godinho *et al.* (2024) introduced the natural proliferative phase protocol (NPP) for FET, in which progesterone LPS is initiated in the late follicular phase, before ovulation, followed by blastocyst transfer on the fifth day of progesterone. When compared to HRT-FET, the NPP protocol proved superior in terms of LBR. Although retrospective, these studies serve as original proof-of-concept, indicating that treatment success might not be as dependent on the natural LH peak and ovulation as previously suspected.

While the efforts to improve the success rates of fertility treatment are important, so are the considerations of patient wellbeing during treatment. In a recently published study, based on a subpopulation of women from the RCT described in this article, Colombo *et al.* (2023) found that use of progesterone LPS in mNC-FET is associated with increased physical discomfort compared to mNC-FET without LPS. Further, using LPS adds extra costs to an already expensive treatment. Conclusively, clinicians are urged to exert caution when deciding whether to use LPS in mNC-FET.

In conclusion, this study found that use of LPS did not significantly increase pregnancy and live birth rates in mNC-FET. Additionally, the study was not able to show an increase in LBR with blastocyst transfer on Day 6 compared to Day 7 after ovulation trigger in mNC-FET cycles. Despite not finding evidence to support our pre-study hypotheses, we believe our results are of great importance. Routine progesterone supplementation is likely unnecessary for most women undergoing FET in an mNC protocol, and more temporal flexibility in timing of blastocyst transfer can be allowed, ultimately paving the way for a simpler, more affordable, and patient-centred approach to FET.

## Supplementary Material

hoag003_Supplementary_Data

## Data Availability

De-identified individual data, including the data dictionary defining each variable in our dataset, as well as code used to analyze data, will be available to researchers upon reasonable request if the research perspectives do not compete with our own plans of publication. We ask that all data-sharing requests be mailed to the lead investigator, A.P. Requests to share data require a detailed description of the study, with objectives and a statistical analysis plan. Data sharing will need approval by the Danish Ethics Committee and the Capital Region of Denmark legal department to ensure compliance in conduct with the General Data Protection Regulation. A data-sharing contract will need to be signed if data sharing is approved.
