# Supplementary material for: Optimizing the protocol for modified natural cycle frozen embryo transfer (mNC-FET): a multicentre, single-blinded randomized controlled trial
Source: Hum Reprod Open. 2026 Jan 13;2026(1):hoag003. doi: 10.1093/hropen/hoag003 (PMC12867578; doi:10.1093/hropen/hoag003)
Supplement: hoag003_Supplementary_Data [file hoag003_supplementary_data.zip › HRO-25-0381-R2-SuppTables1to4.docx]

**Table of contents**

| **Supplementary tables** |  |
| --- | --- |
| Supplementary Table S1 - Pregnancy loss, stratified by type, for women randomised to luteal phase progesterone versus no luteal phase progesterone | 2 |
| Supplementary Table S2 - Pregnancy loss, stratified by type, for women randomised to blastocyst transfer day 6 versus day 7 | 3 |
| Supplementary Table S3 - Reproductive outcomes for women in all study groups in the per-transfer population | 4 |
| Supplementary Table S4 - Adverse obstetric and perinatal outcomes in per-transfer population | 5 |

| **Supplementary Table S1 - Pregnancy loss, stratified by type, for women randomised to luteal phase progesterone versus no luteal phase progesterone** | | | |
| --- | --- | --- | --- |
|  | **Luteal phase progesterone**  **Groups A+B,**  **n (%)** | **No luteal phase progesterone**  **Groups C+D,**  **n (%)** | **P-value^a^** |
| Per-transfer^b^ |  |  |  |
| Total pregnancy loss | 48 (15.9) | 54 (17.9) | 0.59 |
| Early miscarriage | 36 (12.0) | 48 (15.9) | 0.20 |
| Clinical miscarriage | 8 (2.7) | 3 (1.0) | 0.22 |
| Induced abortion | 2 (0.67) | 1 (0.3) | 1.00 |
| Ectopic pregnancy | 2 (0.67) | 2 (0.7) | 1.00 |
| Intention-to-treat^c^ |  |  |  |
| Total pregnancy loss | 48 (15.8) | 54 (17.8) | 0.59 |
| Early miscarriage | 36 (11.8) | 48 (15.8) | 0.20 |
| Clinical miscarriage | 8 (2.6) | 3 (1.0) | 0.22 |
| Induced abortion | 2 (0.7) | 1 (0.3) | 1.00 |
| Ectopic pregnancy | 2 (0.7) | 2 (0.7) | 1.00 |
| Per-protocol^d^ |  |  |  |
| Total pregnancy loss | 46 (15.8) | 53 (18.0) | 0.55 |
| Early miscarriage | 34 (11.6) | 47 (15.9) | 0.17 |
| Clinical miscarriage | 7 (2.7) | 3 (1.0) | 0.14 |
| Induced abortion | 2 (0.7) | 1 (0.3) | 0.62 |
| Ectopic pregnancy | 2 (0.7) | 2 (0.7) | 1.00 |
| As-treated^e^ |  |  |  |
| Total pregnancy loss | 48 (16.0) | 54 (17.9) | 0.60 |
| Early miscarriage | 36 (12.0) | 47 (15.9) | 0.20 |
| Clinical miscarriage | 8 (2.7) | 3 (1.0) | 0.14 |
| Induced abortion | 2 (0.7) | 1 (0.3) | 0.62 |
| Ectopic pregnancy | 2 (0.7) | 2 (0.7) | 1.00 |

^a^For comparisons of binary obstetric complications, we used Chi-squared test (n event >10) or Fisher’s exact test (n events ≤10).

^b^Per-transfer analyses: Included women randomised, undergoing embryo transfer, N=602. Groups A+B, n=301. Groups C+D, n=301.

^c^Intention-to-treat analyses: Included women randomised, N=608. Groups A+B, n=304, Groups C+D, n=304.

^d^Per-protocol analyses: Included women randomised, undergoing allocated treatment, N=587. Groups A+B, n=292. Groups C+D, n=295.

^e^As-treated analyses: Included women randomised, grouped according to treatment received (a total of n=6 women had LPS although they should not according to group allocation), N=601. Groups A+B, n=302. Groups C+D, n=299.

Definitions: Early miscarriage is defined as miscarriage before GA 7+0 (before initial ultrasound scan). Clinical pregnancy loss is defined as miscarriage after initial visualisation of a viable, intrauterine pregnancy by ultrasound.

GA, gestational age.

| **Supplementary Table S2 - Pregnancy loss, stratified by type, for women randomised to blastocyst transfer day 6 versus day 7** | | | |
| --- | --- | --- | --- |
|  | **Transfer day 6**  **Groups A+C,**  **n (%)** | **Transfer day 7**  **Groups B+D,**  **n (%)** | **P-value^a^** |
| Per-transfer^b^ | | | |
| Total pregnancy loss | 53 (17.7) | 49 (16.2) | 0.72 |
| Early miscarriage | 41 (13.7) | 43 (14.2) | 0.93 |
| Clinical miscarriage | 8 (2.7) | 3 (1.0) | 0.14 |
| Induced abortion | 2 (0.7) | 1 (0.3) | 0.62 |
| Ectopic pregnancy | 2 (0.7) | 2 (0.7) | 1.00 |
| Intention-to-treat^c^ |  |  |  |
| Total pregnancy loss | 53 (17.5) | 49 (16.1) | 0.72 |
| Early miscarriage | 41 (13.5) | 43 (14.1) | 0.93 |
| Clinical miscarriage | 8 (2.6) | 3 (1.0) | 0.14 |
| Induced abortion | 2 (0.7) | 1 (0.3) | 0.62 |
| Ectopic pregnancy | 2 (0.7) | (0.7) | 1.00 |
| Per-protocol^d^ | | | |
| Total pregnancy loss | 50 (17.2) | 49 (16.5) | 0.90 |
| Early miscarriage | 38 (13.1) | 43 (14.5) | 0.72 |
| Clinical miscarriage | 8 (2.8) | 3 (1.0) | 0.21 |
| Induced abortion | 2 (0.7) | 1 (0.3) | 0.62 |
| Ectopic pregnancy | 2 (0.7) | 2 (0.7) | 1.00 |
| As-treated^e^ | | | |
| Total pregnancy loss | 50 (17.1) | 52 (16.9) | 1.00 |
| Early miscarriage | 38 (13.0) | 47 (15.3) | 0.49 |
| Clinical miscarriage | 8 (2.7) | 3 (1.0) | 0.19 |
| Induced abortion | 2 (0.7) | 1 (0.3) | 0.62 |
| Ectopic pregnancy | 2 (0.7) | 2 (0.7) | 1.00 |

^a^For comparisons of binary obstetric complications, we used Chi-squared test (n event >10) or Fisher’s exact test (n events ≤10).

^b^Per-transfer analyses: Included women randomised, undergoing embryo transfer, N=602. Groups A+C, n=300. Groups B+C, n=302.

^c^Intention-to-treat analyses: Included women randomised, N=608. Groups A+C, n=303. Groups B+C, n=305.^d^Per-protocol

analyses: Included women randomised, undergoing allocated treatment, N=587. Groups A+C, n=290. Groups B+D, n=297.

^e^ As-treated analyses: Included women randomised, grouped according to treatment received, N=601. Groups A+C, n=293. Groups B+D, n=308.

Definitions: Early miscarriage is defined as pregnancy loss before GA 7+0 (before initial ultrasound scan), including biochemical pregnancies and missed abortions. Clinical miscarriage is defined as pregnancy loss after initial visualisation of a viable, intrauterine pregnancy by ultrasound.

GA, gestational age.

| **Supplementary Table S3 - Reproductive outcomes for women in all study groups in the per-transfer population** | | | | |
| --- | --- | --- | --- | --- |
|  | **Transfer day 6**  **Luteal phase progesterone**  **(n=151),**  **n (%)** | **Transfer day 7**  **Luteal phase progesterone**  **(n=150),**  **n (%)** | **Transfer day 6**  **No luteal phase progesterone**  **(n=149),**  **n (%)** | **Transfer day 7**  **No luteal phase progesterone**  **(n=152),**  **n (%)** |
| Live birth | 50 (35.9) | 52 (34.7) | 46 (30.9) | 50 (32.9) |
| Clinical pregnancy with fetal heartbeat | 60 (39.7) | 55 (36.7) | 49 (32.9) | 51 (33.6) |
| Pregnancy | 78 (51.7) | 75 (50.0) | 74 (49.7) | 76 (50.0) |
| Total pregnancy loss | 25 (16.6) | 23 (15.3) | 28 (18.8) | 26 (17.1) |
| Early miscarriage | 17 (11.3) | 19 (12.7) | 24 (16.1) | 24 (15.8) |
| Clinical miscarriage | 5 (3.3) | 3 (2.0) | 3 (2.0) | 0 |
| Induced abortion | 2 (1.3) | 0 | 0 | 1 (0.7) |
| Ectopic pregnancy | 1 (0.7) | 1 (0.7) | 1 (0.7) | 1 (0.7) |

Definitions: Live birth is defined as birth of a live child following 22 weeks of gestation. Clinical pregnancy is defined as ultrasound verified viable, intrauterine pregnancy after seven weeks of gestation. Pregnancy is defined as serum hCG≥5 IU/ml measured 9-10 days following blastocyst transfer. Early miscarriage is defined as pregnancy loss before GA 7+0 (before initial ultrasound scan), including biochemical pregnancies and missed abortions. Clinical miscarriage is defined as pregnancy loss after initial visualisation of a viable, intrauterine pregnancy by ultrasound.

GA, gestational age.

| **Supplementary Table S4 -** **Adverse obstetric and perinatal outcomes in per-transfer population** | | | | |
| --- | --- | --- | --- | --- |
|  | **Transfer day 6**  **Luteal phase progesterone**  **(n=152)** | **Transfer day 7**  **Luteal phase progesterone**  **(n=152)** | **Transfer day 6**  **No luteal phase progesterone**  **(n=151)** | **Transfer day 6**  **No luteal phase progesterone**  **(n=153)** |
| Congenital malformations | Pyelectasis sin. | Bilateral cryptorchism. | Cystic kidney sin./duplex kidney dxt. | Partial corpus callossum agnesis. |
|  | Hydronephrosis, related to FGFR2-mutation. | Bilateral pyelectasis. |  | Fetal hydrops and increased nuchal fold. |
|  | Twins: One fetus diagnosed with Turner’s syndrome, the other with Down’s syndrome. | Muscular micro-VSD. |  |  |
|  | Giant omphalocele. |  |  |  |
| Perinatal mortality |  | Child died two days following premature delivery at GA 29+0 after placental abruption and pre-eclampsia (same case as had bilateral pyelectasis) |  |  |

GA, gestational age; MTX, methotrexate; VSD, ventricular septal defect.
