# Supplementary material for: Optimizing the protocol for modified natural cycle frozen embryo transfer (mNC-FET): a multicentre, single-blinded randomized controlled trial
Source: Hum Reprod Open. 2026 Jan 13;2026(1):hoag003. doi: 10.1093/hropen/hoag003 (PMC12867578; doi:10.1093/hropen/hoag003)
Supplement: hoag003_Supplementary_Data [file hoag003_supplementary_data.zip › FO_Supplementary_File_S1.pdf]

Preparing and timing of the endometrium in modified natural cycle frozen-thawed embryo transfers (mNC-FET) - a randomized controlled multicenter trial

Statistical analysis plan - Primary outcome paper

Section 1: Administrative information

1. Title and trial registration

1a. Title:

Preparing and timing of the endometrium in modified natural cycle frozen-thawed embryo transfers (mNC-FET) - a randomized controlled multicentre trial

1b. Trial registration:

Registered at ClinicalTrials.gov (NCT03795220) and EudraCT (2018-002207-34).

2-4b. SAP version/Protocol version:

SAP version	Date	Protocol nr	Alterations
**4.0**	15.04.2024	V7	20. As-treated analyses added<br><br>27a. Clarification of statistical methods included in SAP
**3.0**	22.01.2024	V7	7. Reference to protocol paper added<br><br>20. Intention-to-treat and per-protocol analyses added
**2.0**	15.05.2019	V2	19a. Updated to match wording in latest protocol.<br><br>24a-c. Updated to match wording in latest protocol<br><br>29. Additional analyses updated to accommodate inclusion of questionnaires in the trial
**1.0**	23.08.2018	V1	

5. Roles and Responsibilities of contributors to the SAP

Name	Role	Affiliation	SAP contribution
**Marte Saupstad**	Daily primary investigator	Fertility Clinic, Department of Gynaecology, Fertility and Obstetrics, Copenhagen University Hospital -	Prepared initial draft and revisions

		Rigshospitalet, Copenhagen, Denmark	
**Anja Pinborg (PI)**	Sponsor and PI at the main study site	Fertility Clinic, Department of Gynaecology, Fertility and Obstetrics, Copenhagen University Hospital - Rigshospitalet, Copenhagen, Denmark	Reviewed and revised every version
**Kristine Løssl**	Consultant	Fertility Clinic, Department of Gynaecology, Fertility and Obstetrics, Copenhagen University Hospital - Rigshospitalet, Copenhagen, Denmark	Reviewed and revised every version
**Julie Lyng Forman**	Statistical consultant	Section of Biostatistics, Department of Public Health, University of Copenhagen, Copenhagen, Denmark	Reviewed and revised every version

6a-c. Signatures

Person writing the SAP:

Name: Marte Saupstad
Position/title: MD, PhD student

Marte Saupsad 15.04.24

Signature and date

Approved by:

Chief investigator/clinical lead:

Name: Anja Pinborg
Position/title: Professor, DMSc

[illegible] 15.04.2024

Signature and date

Statistician responsible

Name: Julie Lyng Forman
Position/title: Associate professor, PhD

[illegible] 15-04-2024

Signature and date

Section 2: Introduction

7. Background and Rationale

The increasing use of frozen embryo transfer (FET) underscores an urgent need to identify the best protocols for preparing the endometrium in FET Cycles.

The aim of this multicentre RCT is to assess the effect of progesterone supplementation in human choriogonadotropin (hCG) triggered modified natural cycle (mNC) FET and the effect of embryo thawing and transfer at hCG+6 or hCG+7 days, respectively.

A full description of study rationale can be found in the study protocol and protocol paper published in 2019.[1,2]

8. Objectives

(1) To assess if luteal phase support (LPS) in mNC-FET is superior to no LPS in terms of live birth rate per transfer and

(2) To assess if blastocyst warming+transfer 6 days after hCG trigger is superior to warming+transfer 7 days after hCG trigger in terms of live birth rate per transfer in mNC-FET.

Section 3: Study Methods

9. Trial Design

Randomised, single-blinded, controlled, trial using a 2 x 2 factorial design.

Participants will be randomised 1:1:1:1 to the following four study groups:

A) Luteal phase progesterone and warming+transfer 6 days after hCG trigger

B) Luteal phase progesterone and warming+transfer 7 days after hCG trigger

C) No luteal phase progesterone and warming+transfer 6 days after hCG trigger

D) No luteal phase progesterone and warming+transfer 7 days after hCG trigger.

10. Randomisation

"Randomisation will be performed by a study nurse or a non-treating doctor using a randomisation program (dOxos CDS AB) on the day of the hCG trigger.

Randomisation will be performed 1:1:1:1. The randomisation program will take the following confounding factors into account; female age <37 or >=37 years, number of previous oocyte retrievals, number of previous FET cycles, trial site and blastocyst Gardner score at the day of embryo freezing, to secure an equal distribution in the four study arms."[1]

11. Sample Size

"A total of 604 patients are required to have an 80% chance of detecting, at a significance level of 5%, an increase in live birth rate to 31% in the intervention group from 21% in the control group. Sample size calculation does not need correction for the two interventions, as they are considered fully independent of each other."[1]

12. Framework

The study is designed as a superiority trial making the following comparisons:

Comparison I: Vaginal progesterone (group A+B) vs no vaginal progesterone (groups C+D).

Comparison II: Blastocyst warming+transfer day 6 after hCG trigger (group A+C) vs blastocyst warming+transfer day 7 after hCG trigger (group B+D).

13. Statistical Interim Analyses and Stopping Guidance

No interim analyses are planned as it is assumed none of the interventions are at risk of harming the patients' chance of conceiving and giving birth.

14. Timing of Final Analysis

Outcome	Timing of final analysis
**Primary outcome**	
Live birth rate	Following the one-year follow-up of last patient enrolled in the trial with a verified clinical pregnancy and no record of pregnancy loss (one-year follow-up)
**Secondary outcomes**	
Pregnancy rate	When the last patient participating in the trial has completed her pregnancy test
Clinical pregnancy rate	When all patients enrolled I the trial, with a positive pregnancy test, have attended their early pregnancy scan
Pregnancy loss rate	Following the one-year follow-up of last patient enrolled in the trial with a verified clinical pregnancy and no record of pregnancy loss (one-year follow-up)
Obstetric and perinatal outcomes	Following the one-year follow-up of last patient enrolled in the trial with a verified clinical pregnancy and no record of pregnancy loss (one-year follow-up)

15. Timing of Outcome Assessments

All outcomes will be assessed at an individual level.

Outcome	Timing of outcome assessments
**Primary outcome**	
**Live birth rate**	Approximately one year after inclusion in trial (one-year follow-up) for all patients with a verified clinical pregnancy and no record of pregnancy loss
**Secondary outcomes**	
**Pregnancy rate**	Assessed following the pregnancy test conducted at ovulation trigger + 16 days.
**Clinical pregnancy rate**	Assessed at the early pregnancy scan planned in gestational week 8 of all patients with a positive pregnancy test
**Pregnancy loss rate**	Assessed continuously in patients with a positive pregnancy test, depending on the time point of pregnancy loss
**Obstetric and perinatal outcomes**	Approximately one year after inclusion in trial (one-year follow-up) for all patients with a verified clinical pregnancy and no record of pregnancy loss

Section 4: Statistical Principles

Confidence Intervals and P-Values

16. Level of statistical significance to be used.

A significance level of 5% will be used.

17. Adjustment for Multiplicity

No adjustment for multiplicity will be conducted.

18. Confidence Intervals

95% confidence intervals for mean differences, risk differences (RD) and adjusted risk differences (aRD) will be calculated.

19. Adherence and Protocol Deviations

19a. Definition and assessment of adherence.

Adherence to study interventions will be logged continuously throughout the trial.

Comparison I: Use of LPS vs no use of LPS

"Complete medicine accounts will be kept by the study nurses at each of the trial sites. All medication handed out to the patients will be recorded and patients are instructed to inform the study personnel about deviations in medicine administration and to deliver all medication packings, with or without remaining medicine, back to the study nurses after end of participation.

The study nurses at Rigshospitalet will keep the overall medicine accounts files of the study medication used in the project and collect all the medicine accounts from each of the trial sites after end of inclusion."[1]

Comparison II: Timing of blastocyst transfer

Due to the nature of the intervention, it will not be possible for patients to deviate from the allocated intervention, as their blastocyst will be thawed, and blastocyst transfer scheduled, at the allocated day of blastocyst transfer. It might however happen that a participating study clinic cannot offer blastocyst transfer on the allocated day due to weekly closing days or bank holidays. In this case, the patient will be offered treatment on the previous or next day according to local routines. These patients will be included in the per-transfer, intention-to-treat and as-treated analyses, but excluded from the per-protocol analyses.

19b. Presentation of adherence data.

If gross inability to follow allocated study group is detected, data will be presented in publication reporting on the primary outcome.

It is expected that some patients will forget to administer one-several doses of their LPS during the trial, seeing as the medication is administered three times daily. While all deviations will be accounted for by the participating study clinics, minor deviations in adherence to study interventions will not be publicly reported.

19c. Definition of protocol deviations.

All deviations from protocol will be reported and registered in the online case report form (CRF)/study database. Minor deviations may include, but is not limited to missingness of blood samples, study visits planned at day not suggested in protocol and errors during randomisation.

Major protocol deviations (patient or clinic not being able to comply with allocated study group) will be reported to the primary investigator (PI) continuously via the CRF.

19d. Summary and presentation of protocol deviations.

AP (PI), KL and MS will assess protocol deviations continuously during the trial. Minor protocol deviations will not be publicly presented. Major protocol deviations will be reported in the primary outcome paper.

20. Analysis Populations

Per-transfer (primary population): Women randomised, undergoing embryo transfer

Intention-to-treat: Women randomised.

As-treated: Women randomised, grouped according to treatment received.

Per-protocol: Women randomised, undergoing allocated treatment.

Women withdrawing consent to participate in the trial will be removed from all analyses.

Section 5: Trial Population

21. Screening Data

Screening data will not be reported due to Danish legislation not allowing for collection of data on women not consenting to trial participation.

22. Eligibility

"Women fulfilling following criteria:

- Age 18-41 years
- Regular menstrual cycle (23-35 days)
- Planned to undergo single blastocyst transfer in a modified natural cycle with a high-quality (Gardner 3-6 A/B), vitrified blastocysts derived from 1.-3. IVF/ICSI cycle resulting in an embryo transfer on a public hospital.
- Giving informed consent

Exclusion criteria:

- Previous participation in the study
- Uterine malformations, intrauterine polyps or submucosal myomas
- Current breast feeding
- Receiving oocyte donation
- Preimplantation genetic testing for heritable diseases
- Blastocyst conceived with sperm from testicular sperm aspiration
- Active HIV or hepatitis B and C infection
- Known luteal phase insufficiency

Further exclusion criteria are the following contraindications to progesterone: allergy to the study medication, undiagnosed vaginal bleeding, current missed abortion or ectopic pregnancy, hepatic insufficiency or severe hepatic disease, history of genital or breast cancer, history of arterial or venous thromboembolism, thrombophlebitis or porphyria."[1]

23. Recruitment

Patients scheduled to start FET treatment in a mNC will be recruited continuously through oral and written communication.

A standard CONSORT diagram will be made with exception of the number of women screened for participation.[3] Danish GDPR legislation does not allow for collection of data on women not giving consent to participate in the trial.

Withdrawal/Follow-Up

24a-c. Levels and reasons for withdrawal or loss to follow-up.

"A patient can withdraw from the study at any time. Further, participation in the study can be interrupted by the non-treating and the treating doctors, if one of the following criteria is present:

• The patients general condition contraindicates participation

• Protocol violation, which the investigator considers having influence on the treatment"[1]

We do not expect patients to be lost to follow-up during the fertility treatment course or during pregnancy. Home-birth is not common in Denmark, still, information regarding potential obstetric complications and perinatal outcomes might be lost should a patient decide to give birth without involvement of the public health sector.

In the case of missing outcome data, this will be publicly disseminated in the primary outcome paper.

25. Baseline Patient Characteristics

25a-b. List of baseline characteristics to be summarized and methods for summarizing baseline characteristics

Baseline characteristics to be summarized	Unit	Method for summarizing baseline characteristics
Age	Years	Means (SD) or median (IQR) depending on the distribution
Age ≥37 years	Years	n (%)
Age ≥35 years	Years	n (%)
Ethnicity	Caucasian, African, Afro-Caribbean, Asian, Inuit, other.	n (%)
Civil status	Heterosexual, homosexual or single.	n (%)

Body mass index	Kg/m^2	Means (SD) or median (IQR) depending on the distribution
Smoking daily	Yes/no	n (%)
Menstrual cycle length	Days	Means (SD) or median (IQR) depending on the distribution
Duration of infertility	Years	Means (SD) or median (IQR) depending on the distribution
Antral follicle count	N	Means (SD) or median (IQR) depending on the distribution
AMH	pmol/L	Means (SD) or median (IQR) depending on the distribution
Cause of infertility	Anovulation, tubal factor, cervical uterine factor, endometriosis, male factor, unexplained, other.	n (%)
Number of IVF/ICSI cycles	1, 2, 3	n (%)
Number of FET cycles	1, 2, ≥3	n (%)
Previous childbirth	Yes/no	n (%)
Previous pregnancy	Yes/no	n (%)

SD = standard deviation, IQR = interquartile range, AMH = anti-Müllerian hormone, IVF = in vitro fertilisation, ICSI = intracytoplasmic sperm injection, FET = frozen embryo transfer

**Treatment characteristics to be summarized**	**Unit and**	**Method for summarizing baseline characteristics**
Cycle day of ovulation trigger	Cycle day	Means (SD) or median (IQR) depending on the distribution
Size of leading follicle at trigger	mm	Means (SD) or median (IQR) depending on the distribution
Endometrial thickness at trigger	mm	Means (SD) or median (IQR) depending on the distribution

Method of fertilitsation of blastocyst rated best at time of inclusion	IVF/ICSI	n (%)
Blastocyst Gardner score	3AA/AB/BA/BB, 4AA/AB/BA/BB, 5-6AA/AB/BA/BB	n (%)
Day of blastocyst vitrification	Day 5/ day 6	n (%)

SD = standard deviation, IQR = interquartile range, IVF = in vitro fertilisation, ICSI = intracytoplasmic sperm injection

Section 6: Analysis

Outcome Definitions

26a-b. Specification, unit, measurement method and timing of primary and secondary outcomes

Outcome	Definition	Unit	Method of measurement	Time of evaluation
**Primary outcome**				
Live birth	Defined as live birth following GA 22+0 weeks.	Yes/no	Registration in medical records	Approximately one year after inclusion in trial (one-year follow-up) for all patients with a verified clinical pregnancy and no record of pregnancy loss
**Secondary outcomes**				
Clinical pregnancy	Intrauterine embryo with fetal heartbeat detected at the early pregnancy scan.	Yes/no	Vaginal ultrasound	Assessed at the early pregnancy scan planned in gestational week 8 of all patients with a positive pregnancy test
Pregnancy	s-hCG ≥ 5 IU/L	Yes/no	Blood sample	16 days following ovulation trigger
Pregnancy loss	All cases of pregnancy loss follow-	Yes/no	Registration in medical records or detection of empty	Assessed continuously in patients with a positive pregnancy

	ing a positive pregnancy test, before GA 22+0		uterus/fetal demise at early pregnancy scan	test, depending on the time point of pregnancy loss
Ectopic pregnancy	Pregnancy detected outside of the uterus, using either a) ultrasound or b) histologic testing	Yes/no	Registration in medical records or detection of ectopic pregnancy during the early pregnancy ultrasound scan	Assessed continuously in all patients with a positive pregnancy test
Induced abortion	Medically or surgically induced abortion, typically due to congenital abnormality in fetus/child	Yes/no	Registration in medical records	Assessed continuously in all patients with a positive pregnancy test
Early miscarriage	Pregnancy loss before vitrification of a clinical pregnancy	Yes/no	Registration in medical records	Assessed continuously in all patients with a positive pregnancy test
Late miscarriage	Pregnancy loss before vitrification of a clinical pregnancy, before GA 22+0	Yes/no	Registration in medical records	Assessed continuously in all patients with a positive pregnancy test
**Obstetric complications**				Approximately one year after inclusion in trial (one-year follow-up) for all patients with a verified clinical pregnancy and no record of pregnancy loss
Hypertensive disorders of pregnancy	Including pre-eclampsia, eclampsia and gestational hypertension	Yes/no	Registration in medical records	

Gestational diabetes	Diabetes detected during pregnancy with oral glucose tolerance test.	Yes/no	Registration in medical records	
Postpartum bleeding	Bleeding exceeding 1000 mL	Yes/no	Registration in medical records	
Caesarean section		Yes/no	Registration in medical records	
**Perinatal complications**				Approximately one year after inclusion in trial (one-year follow-up) for all patients with a verified clinical pregnancy and no record of pregnancy loss
Preterm birth	Birth before GA 37+0	Yes/no	Registration in medical records	
Birthweight		Grams	Registration in medical records	
Low birth weight	Birthweight < 2500 grams	Yes/no	Registration in medical records	
Very low birth weight	Birthweight < 1500 grams	Yes/no	Registration in medical records	
Hight birth weight	Birthweight > 4000 grams	Yes/no	Registration in medical records	
Very high birth weight	Birthweight > 4500 grams	Yes/no	Registration in medical records	

Small for gestational age	More than two standard deviation units below expected birth weight.	Yes/no	Calculated following collection of birth-weight data	
Large for gestational age	More than two standard deviation units above expected birth weight.	Yes/no	Calculated following collection of birth-weight data	
Congenital malformation	Any congenital malformation	Yes/no	Registration in medical records	
Perinatal death	Stillbirth or live birth followed by death within the first week following birth, from GA 22+0	Yes/no	Registration in medical records	

GA = gestational age, hCG = human chorionic gonadotropin

26c. Calculations or transformations applied to outcomes.

In the final analysis the rate of the above outcomes will be calculated using the formula: n events / N individuals in study group in a given study population supplied by percentages.

Analysis Methods

27a. Statistical methods for each outcome and presentation of treatment effects.

"For comparisons between the study groups we will use Student's t-test for continuous variables and in case of non-parametric data Kruskal-Wallis test. For comparisons of proportions, we will use Chi-squared tests. For the adjusted analyses we will use multiple linear and logistic regression analyses for continuous endpoints and proportions respectively."[1]

Clarification:

The results of from the logistic regression analyses will be presented as adjusted risk differences to better assess the clinical relevance of the potential treatment effects.

27b. Adjustments for covariates.

The multivariable regression analyses will be adjusted for the stratification variables (trial site, female age <37 or >=37 years, number of previous oocyte retrievals, number of previous FET cycles, blastocyst expansion grade at the day of embryo freezing) in addition to parity and day of blastocyst vitrification.[4]

27c. Assumptions checks for statistical methods.

Not relevant.

27d. Alternative methods if assumptions are violated.

For comparisons of binary obstetric complications and perinatal outcomes, we will use Fisher's exact test, instead of the Chi-square test, if the number of events is ≤ 10.

27e. Planned sensitivity analyses

Not relevant.

27f. Planned subgroup analyses and definitions

Raw count data describing the distribution of reproductive outcomes per study group (A, B, C and D) will be presented in Supplementary files. Comparisons of reproductive outcomes between separate study groups will not be conducted.

28. Missing Data

For transparency, rates will be presented as a fraction (e.g. n events/n individuals in study group) and percentage.

29. Additional Analyses

Analyses on the association between the level of reproductive hormones on the day on ovulation trigger and on the day of blastocyst transfer with reproductive outcomes will be performed and published in separate publications. Analyses and outcomes described in said paper(-s) will not be further described in this statistical analysis plan.

Analyses on the self-reported health and wellbeing of women participating in the clinical trial and use of luteal phase progesterone support will also be conducted. Analyses and outcomes described in said paper(-s) will not be further described in this statistical analysis plan.

30. Harms

Progesterone is routinely used for patients undergoing ovarian stimulation and embryo transfer and is not expected to cause harm to participants. The timing of embryo transfer will not cause any harm to the patients. Apart from extra study visits and blood samples, this study will cause very modest harm to participants.

In case of adverse reactions (AR), serious adverse events (SAEs), serious adverse reactions (SARs) and suspected unexpected serious adverse reactions (SUSARs) these will be registered

by allocated study staff according to study protocol. Data on patient safety and potential SAEs, SARs and SUSARs will be reported to the Danish Medicines agency annually according to Danish law.

Should patients participating in the trial give birth to children with congenital malformations or in the rare event of perinatal death, these outcomes will be publicly disseminated under "perinatal outcomes", including comparisons of these outcomes across study groups.

Statistical Software

31. Details on statistical packages

R Studio will be used to conduct the statistical analyses.

References

1 Saupstad M, Løssl K, Pinborg AB. Study protocol for 'Preparing and timing of the endometrium in modified natural cycle frozen-thawed embryo transfers (mNC-FET) - a randomized controlled multicenter trial'. 2024.

2 Saupstad M, Freiesleben NLC, Skouby SO, *et al*. Preparation of the endometrium and timing of blastocyst transfer in modified natural cycle frozen-thawed embryo transfers (mNC-FET): A study protocol for a randomised controlled multicentre trial. *BMJ Open*. 2019;9:1–7. doi: 10.1136/bmjopen-2019-031811

3 Schulz KF, Altman DG, Moher D. CONSORT 2010 statement: Updated guidelines for reporting parallel group randomised trials. *International Journal of Surgery*. 2011;9:672–7. doi: 10.1016/j.ijsu.2011.09.004

4 Kahan BC, Morris TP. Improper analysis of trials randomised using stratified blocks or minimisation. *Stat Med*. 2012;31:328–40. doi: 10.1002/sim.4431
